# Global Corticospinal Excitability as Assessed in A Non-Exercised Upper Limb Muscle Compared Between Concentric and Eccentric Modes of Leg Cycling

**DOI:** 10.1038/s41598-019-55858-5

**Published:** 2019-12-16

**Authors:** Joel A. Walsh, Paul J. Stapley, Jonathan B. H. Shemmell, Romuald Lepers, Darryl J. McAndrew

**Affiliations:** 1 Neural Control of Movement Laboratory, School of Medicine, Faculty of Science, Medicine and Health, University of Wollongong, New South Wales, Australia; 2 Neuromotor Adaptation Laboratory, School of Medicine, Faculty of Science, Medicine and Health, University of Wollongong, New South Wales, Australia; 3 INSERM UMR1093-CAPS, Université Bourgogne Franche-Comté, UFR des Sciences du Sport, Dijon, France; 4 Discipline of Graduate Medicine, School of Medicine, Faculty of Science, Medicine and Health, University of Wollongong, New South Wales, Australia; 5 Illawarra Health and Medical Research Institute (IHMRI) University of Wollongong, New South Wales, Australia

**Keywords:** Neurophysiology, Translational research

## Abstract

This study investigated the effects of eccentric (ECC) and concentric (CON) semi-recumbent leg cycling on global corticospinal excitability (CSE), assessed through the activity of a non-exercised hand muscle. Thirteen healthy male adults completed two 30-min bouts of moderate intensity ECC and CON recumbent cycling on separate days. Power output (POutput), heart rate (HR) and cadence were monitored during cycling. Global CSE was assessed using transcranial magnetic stimulation to elicit motor-evoked potentials (MEP) in the right first dorsal interosseous muscle before (‘Pre’), interleaved (at 10 and 20 mins, t10 and t20, respectively), immediately after (post, P0), and 30-min post exercise (P30). Participants briefly stopped pedalling (no more than 60 s) while stimulation was applied at the t10 and t20 time-points of cycling. Mean POutput, and rate of perceived exertion (RPE) did not differ between ECC and CON cycling and HR was significantly lower during ECC cycling (*P* = 0.01). Group mean MEP amplitudes were not significantly different between ECC and CON cycling at P0, t10, t20, and P30 and CON (at *P* > 0.05). Individual participant ratios of POutput and MEP amplitude showed large variability across the two modes of cycling, as did changes in slope of stimulus-response curves. These results suggest that compared to ‘Pre’ values, group mean CSE is not significantly affected by low-moderate intensity leg cycling in both modes. However, POutput and CSE show wide inter-participant variability which has implications for individual neural responses to CON and ECC cycling and rates of adaptation to a novel (ECC) mode. The study of CSE should therefore be analysed for each participant individually in relation to relevant physiological variables and account for familiarisation to semi-recumbent ECC leg cycling.

## Introduction

It is known that both regular and single bouts of exercise can enhance cortical plasticity and excitability^1–3^. In particular, stationary leg cycling involving concentric (CON) or shortening muscle contractions, is known not only to enhance intracortical excitability in the muscles involved in the exercise, but also in the muscles not directly involved in pedalling, such as wrist muscles^4^. This is supported by the work of Zehr and colleagues, who showed that motor evoked potentials (MEPs) elicited in non-exercising arm muscles are facilitated *during* submaximal leg cycling^5^. Similarly, *during* low-intensity cycling using the arms, MEPs measured in the non-exercising vastus lateralis (leg) muscle also increased^6^. Taken together, these findings indicate that as facilitation was observed in non-exercising muscles, exercise contributes to increasing a state of ‘global corticospinal excitability’ (CSE). These results also suggest a neural coupling between the upper and lower limbs. Moreover, Zehr’s work suggests that as well as descending (cortical) commands, spinal mechanisms (e.g., central pattern generators; CPGs) also contribute to the control of rhythmic arm or leg movements^5,7^. Furthermore,^8^ recorded greater CSE in non-exercised wrist muscles after cycling with the legs when compared to static contractions of the same leg muscles. Moreover, these authors also showed that sub-threshold transcranial magnetic stimulation (TMS) facilitated H-reflex amplitudes during cycling and static contractions to the same extent, suggesting that sub-cortical sites likely contribute to the increase in global CSE seen in the non-exercised muscle.

Cycling exercise is known to reduce cardiovascular risk factors^9^, improve functional muscle performance^10^ and enhance cognitive function^4^. Two modes of cycling have been increasingly used in rehabilitation settings: (1) the regular, concentric (CON) mode, in which active muscles shorten during contractions, or (2) an eccentric (ECC) mode, during which participants contract active leg muscles while they lengthen under a load applied by the pedals being driven in a backwards direction. Differences in neuromuscular activity have been reported between submaximal single-leg CON and ECC cycling exercise^11,12^. For a given workload, ECC cycling increases muscle strength and mass at a significantly reduced cardiovascular cost compared to CON cycling^13–16^. This benefit has led to ECC cycling being adopted as a form of clinical rehabilitation among patients to minimise cardiovascular stress^17–19^.

While there are clear advantages of using ECC cycling with clinical populations (improving strength at lower cardiovascular loads), whether this mode of cycling is associated with neural plasticity, and more specifically changes in global CSE, is unknown. What evidence is there that ECC cycling would result in quantitatively different measures of global CSE during or after cycling, compared to CON cycling?^4^ quantified global CSE (input-output curves of MEPs) using a non-exercised hand muscle before, immediately after and 30 min post CON cycling in a recumbent position. In addition, they measured short and long intracortical inhibition (SICI and LICI, respectively) and intracortical facilitation (ICF). Although they found no differences in CSE at different stimulus intensities before, immediately after or 30 min post exercise, they did find that CON cycling for 20 min led to increased ICF and decreased SICI. Their results therefore contrast somewhat with those of^8^ who recorded greater global CSE in non-exercised muscles after regular CON leg cycling than static contractions of the same muscles as those used during cycling. On the contrary, ECC muscle contractions have been associated with reduced CSE due to the inhibition of Ia afferents, as well as recurrent inhibition to regulate gain at the spinal level^20,21^. Moreover, H-reflex activity has been shown to be depressed during ECC muscle contraction, suggesting that spinal mechanisms are modulated by supra-spinal structures^22^. Greater cortical motor control is required to execute ECC muscle contractions^23–25^ suggesting that ECC exercise, including ECC cycling is modulated by different supra-spinal neural control strategies, compared to CON cycling. Therefore, the aim of the current study was to determine how low-intensity semi-recumbent ECC and CON leg cycling affects global CSE as measured in a non-exercising hand muscle. Our study adopted an exercise intensity of cycling ergometry (low-moderate) previously used to study muscle strength in with clinical populations^18^. We predicted that, semi-recumbent CON cycling would show facilitation of CSE, whereas semi-recumbent ECC cycling (associated with spinal inhibition) would not show such facilitation.

## Materials and Methods

### Participants

Thirteen healthy male participants (*n* = 13: age: 24.3 ± 3.5 years, weight: 82.2 ± 10.5 kg, height: 180.1 ± 6.6 cm, mean ± SD) with no history of medical or neurological disorders volunteered to participate in the study. An estimated sample size of six participants calculated using an α-level of 0.05, power (1 – β) of 0.80 and an effect size of (Cohen’s *d*) 0.76^26^. Participants completed a Sports Medicine Australia pre-exercise screening questionnaire to determine exercise readiness. Self-reported physical activity levels were calculated using the Long Format International Physical Activity Questionnaire^27^. Eleven of 13 participants were right handed based on Edinburgh Handedness Inventory scores (mean LQ = 83.6 ± 24.7%). All participants provided informed consent prior to participation, with the study being approved by the University’s Human Research Ethics Committee and carried out in accordance with the Declaration of Helsinki.

### Experimental design

**Figure 1:**
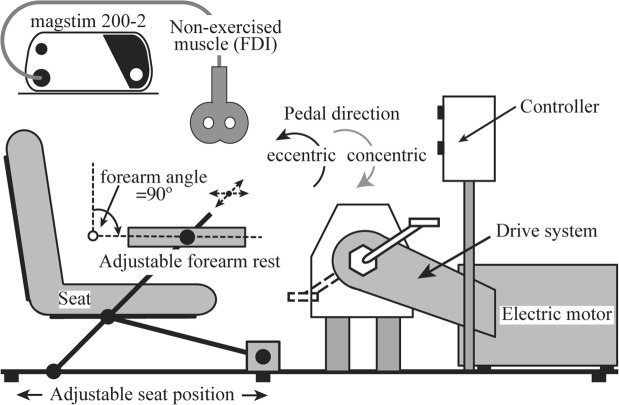
Experimental set-up. Participants (*n* = 13) were seated in a semi-recumbent cycle able to function under two modes: concentric (CON) and eccentric (ECC). The right forearm was placed on an adjustable arm rest such that the forearm was immobilised with a fixed elbow joint angle of 90° (palm pronated) on an arm-rest.

Participants were randomly allocated to two intervention orders (ECC then CON or CON then ECC, Fig. 1), separated by 24–96 hours, in a cross-over design. Prior to ECC cycling only, participants performed five-minutes of ECC cycling at a workload of 1 W/kg to become familiarised with the reverse (i.e. backwards) motion of semi-recumbent ECC leg cycling^28–30^. Participants completed 30 minutes of low-intensity CON or ECC recumbent leg cycling (3 × 10 min bouts). Power output (POutput) and heart rate (HR) were collected throughout the full duration of the experiments. Ratings of perceived exertion (RPE) and TMS-derived MEPs were measured before leg cycling and at 10 min intervals during the 30 mins (times hereon referred to as ‘interleaved’) and post exercise. Heart rate is reported in 7/13 participants as the remaining five participants demonstrated interference in the HR signal and did not have complete datasets. Participants remained seated for the duration of testing and all non-cycling movement was minimised by asking participants to hold their arm crossed in their laps. Participants were instructed to use the first 30 s of cycling to stabilize their self-perceived intensity at a set cadence (60 rpm).

### Cycle ergometer

**Figure 2:**
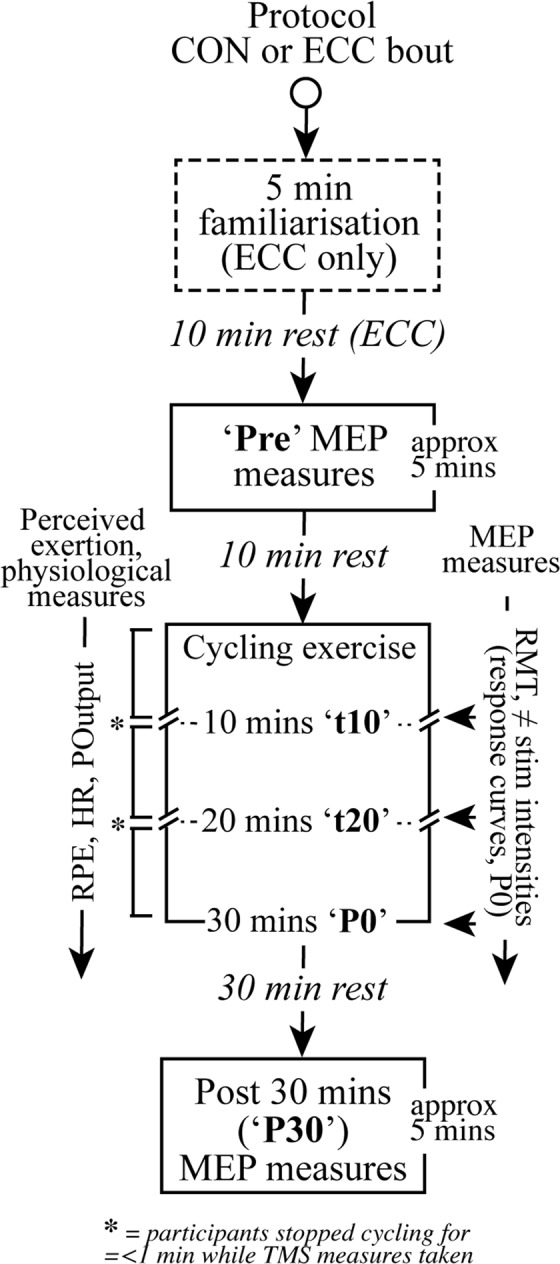
Cycling protocol and timeline of TMS measurements for ECC and CON experimental sessions (2 sessions per participant). Resting motor threshold (RMT) was collected at ‘Pre’, t10, t20, P0 and P30. Stimulus-response curves were collected at ‘Pre’ and P0. As indicated by *, participants stopped cycling (referred to as interleaved periods in the text) for a short period not exceeding 60 s at after 10 mins during each 30 min cycling bout.

All cycling exercises were carried out on a custom-built recumbent cycle ergometer that restricted muscle contraction of the lower limb to either ECC or CON within a closed-chain loop (Fig. 2). For CON cycling, an existing magnetic braked regulation box (Siemens, CAmed, Germany) provided resistance, while for ECC cycling; a 0.75 kW asynchronous electric motor drove the cranks backwards with participants resisting the pedal movements. Participants were seated to achieve an extended knee angle of 130° (relative angle)^11^. A voltage control dial connected to the magnet and motor-controlled resistance that could be adjusted in 10 or 20 W increments. Power output was measured using a Schoberer Rad Meßtechnik (SRM) PowerCrank system (Julich, Germany). Zero offset of the SRM PowerCrank system was carried out prior to each testing session. Participants were instructed to maintain a cadence (rpm) of 60 rpm by watching the SRM head unit (SRM PowerControl V7) and adjusting the voltage control dial to maintain a self-perceived level of cycling resistance. Heart rate data was collected continuously throughout each CON and ECC cycling session using a Suunto Dual Comfort Belt (Suunto Oy, Vantaa, Finland).

### Exercise intensity

Participants were required to maintain a low-moderate intensity cycling workload, similar to that adopted in a previous study (moderate; RPE 10-12 or CR-10 of 3; MacMillan, 2017) using the Borg 6–20 rate of perceived exertion (RPE) scale^31^. All participants had prior experience using the Borg scale. Participants reported their RPE every two minutes to confirm the stability of perceived cycling intensity and it was recorded every 10^th^ min (i.e. t10, t20 and P0; Fig. 1).

### Electromyography

Surface electromyography (EMG) was recorded from the right (dominant) first dorsal interosseous (FDI) muscle using 10 mm diameter Ag/AgCl bipolar electrodes fixed in a belly-tendon montage. Prior to electrode placement the skin was prepared by shaving, mildly abrading and cleansing with isopropyl alcohol. The location of the EMG electrode was traced onto the skin of each participant so that it could be replaced in the same position during the following visit. EMG signals were sampled at 1 kHz (Power1401, Cambridge Electronic Design, Cambridge, UK) gain amplified (×1000) and bandpass filtered (20 Hz–1000 Hz). Offline analysis of EMG data was performed using Spike software version 6.02 (Cambridge Electronic Design, Cambridge, UK).

### Ulnar nerve stimulation

Five maximal M-wave (M_max_) recordings were taken from the FDI prior to pre-exercise TMS measures. To evoke M_max_ in FDI, single square-wave stimulation currents of 200 µs duration were delivered to the ulnar nerve using a bar electrode (MLADDF30 stimulating bar electrode, ADInstruments, Bella Vista, Australia) placed 3 cm proximal to the wrist^2,32^. Stimulator output (DS7AH, Welwyn, Garden City, UK) was incrementally increased until M-wave plateaued^32^. Stimulator output where the M-wave plateaued was considered as the plateau intensity (100%; mean stimulator intensity; 44.5 ± 9.6 mA). Supramaximal stimulations equating to 120% of an individual’s plateau intensity were delivered to evoke M_max_ (mean stimulator intensity; 53.4 ± 11.5 mA).

### Transcranial magnetic stimulation

Single pulse TMS was delivered over the primary motor cortex (M1) region using a Magstim 200 (Magstim Co. Ltd, UK) and a figure-8 coil (70 mm diameter). The coil was aligned tangentially to the sagittal plane at a 45° angle over the hand region of the left M1 to induce a posterior to anterior current flow within the motor cortex. Optimal coil position (hotspot) was determined as the location and orientation that evoked the largest MEPs in the relaxed right FDI. Optimal coil position was marked directly on a fitted cap for accurate repositioning throughout each testing session.

### Motor-evoked potentials

Resting motor threshold (RMT) was determined as the lowest stimulus intensity that would elicit at least 5/10 MEPs^33^ with a peak-to-peak amplitude >50 V^34^ in the relaxed right FDI. Hand and finger position was demarcated on the arm-rest to allow for accurate repositioning of the hand and fingers in order to maintain consistent FDI muscle length and orientation. Each participant had their right arm positioned at an elbow joint angle of 90° (palm pronated) on an arm-rest, while TMS was administered (Fig. 2). Ten MEPs (ISI = 4–5 s; RMT = ECC; 48.1 ± 5.9, CON; 48.5 ± 6.9% MSO) were elicited in the right FDI pre-exercise (‘Pre’), interleaved (t10, t20), immediately post exercise (P0) and 30 min post-exercise (P30). For RMT measurements of the right FDI taken at 10 and 20, participants briefly stopped cycling on the ergometer (no longer than 60 s). Stimulus-response curves (SRC) were generated using single-pulse TMS to elicit MEPs in the resting FDI muscle at four intensities based on motor threshold (five stimuli per intensity): 90%, 100%, 110% and 120% RMT. Intensities were randomised across participants in a blocked order at ‘Pre’ and P0 times, remaining consistent within the testing session. Peak-to-peak MEP amplitudes were calculated from the region of EMG activity as per procedures of^35^. All MEP amplitudes were normalised to M_max_ in order to provide an indication of the percentage of the motoneuron pool recruited during MEPs and minimise inter-participant variability due to differences in impedance, electrode placement relative to innervation points, muscle architecture, etc, between subjects.

### Statistical analysis

The IBM SPSS Statistics for Windows, version 21 (IBM Corp., Armonk, N.Y., USA) software was used for all statistical analyses. Paired *t* tests were used to compare ECC and CON RPE, relative power (W/kg^−1^), cadence and heart rate (HR). Data are presented as mean ± standard deviation (SD). All MEP data were tested for normality using a Shapiro-Wilk analysis and sphericity using Mauchly’s sphericity test prior to statistical analysis. Where sphericity was violated (significant, *P* > 0.05; F-ratio invalid) Greenhouse-Geisser corrections (ε < 0.75) were used. Effect size for post-hoc tests and analysis of variance are reported using Cohen’s *d* and partial ETA squared values (η_p_^2^).

Peak-to-peak mean RMT MEP amplitudes were analysed using a two-way repeated analysis of variance (RM-ANOVA) using TIME (Pre, t10, t20, P0 and P30) and EXERCISE (ECC and CON) as within-subject factors. Mean peak-to-peak amplitudes of SRC MEPs were analysed using a three-way RM-ANOVA with TIME (Pre, P0 and P30), EXERCISE (ECC and CON) and STIMULUS INTENSITY (90, 100, 110 and 120% RMT) as within-subject factors. Post-hoc analysis of parametric data was carried out using Bonferroni adjustments. Mean MEP amplitude responses at 90%, 100%, 110% and 120% of RMT were correlated using Pearson’s coefficient (*r*) between ECC and CON cycling and reported alongside the mean slope of the SRC. Individual peak-to-peak SRC MEP amplitudes were compared to respective ‘Pre’, using one-way ANOVA. Relative difference between individual slopes of the SRC were calculated for CON and ECC cycling modes by dividing the MEP amplitude by the stimulus intensity and subtracting ‘Pre’ and P0 slope values. Post Hoc analysis of homogenous individual MEP amplitude data was carried out using Tukey’s HSD test, Welch test with Games-Howell post-hoc analysis was carried out on non-homogenous data. All MEP data were normalised to pre-exercise M_max_ amplitude^36,37^ and presented as mean ± standard deviation (SD) or mean ± standard error of the mean (SEM) with significance set at *P* ≤ 0.05.

## Results

All participants achieved physical activity levels greater than 600 metabolic equivalents (METs) and were therefore deemed to be moderately physically active. No significant differences in mean relative POutput (ECC; 0.97 ± 0.2, CON; 1.00 ± 0.3 W/kg^−1^, *P* = 0.49, *d* = 0.16), cadence (ECC; 57.2 ± 6.6, CON; 61.3 ± 7.2 rpm, *P* = 0.06, *d* = 0.60) or RPE (ECC; 11.3 ± 0.8, CON; 11.3 ± 0.8, *P* = 0.70, *d* = 0.14) were observed between ECC and CON modes of cycling. However, for the same POutput and RPE, HR was significantly lower during ECC cycling compared to CON cycling (ECC; 72.5 ± 6.7, CON; 96 ± 4.3 bpm, *P* = 0.01, *d* = 4.21).

### Cycling power output

**Figure 3:**
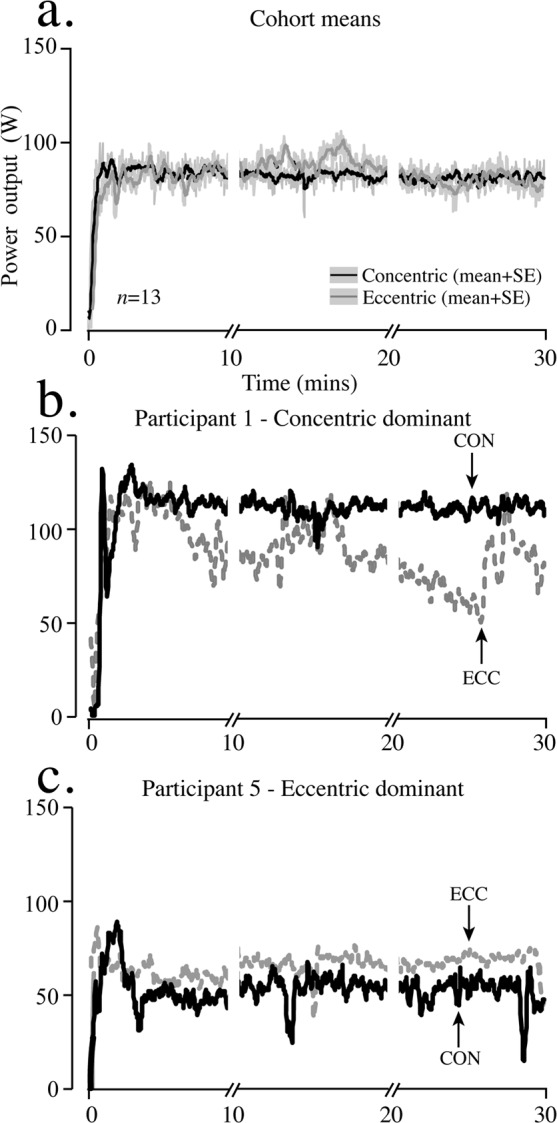
Cycling power output profiles. (**a**) Mean (±Standard error) power output profiles for 30 min of low-intensity ECC (dotted) and CON (bold) semi-recumbent cycling completed at an average cadence of 57 rpm. (**b**) A participant representing that where POutput was higher during CON than ECC cycling (*n* = 8). (**c**) A participant in which POutput was higher during ECC compared to CON cycling (*n* = 5). Cessation periods (i.e. interleaved) of cycling at t10 and t20 (<90 s) have been removed from power profiles to display continuous POutput for 30-min of recumbent cycling.

Mean POutput was not different between CON and ECC cycling for the entire period or at each timepoint (t10, t20 and P0; Fig. 3a). Even though the range of mean POutput was smaller at 10, 20, and P0 and for the entire bout for ECC compared to CON cycling at a set cadence of 60 rpm (cohort mean across modes were 59 ± 3.7 rpm), participants showed higher power profiles in CON or ECC conditions. For example, Fig. 3b shows a participant who produced constant and higher CON POutput and a lower, more stochastic ECC POutput profile. Alternatively, Fig. 3c shows a participant who produced consistently higher POutput in ECC compared to CON cycling.

### Motor-evoked potentials

**Figure 4:**
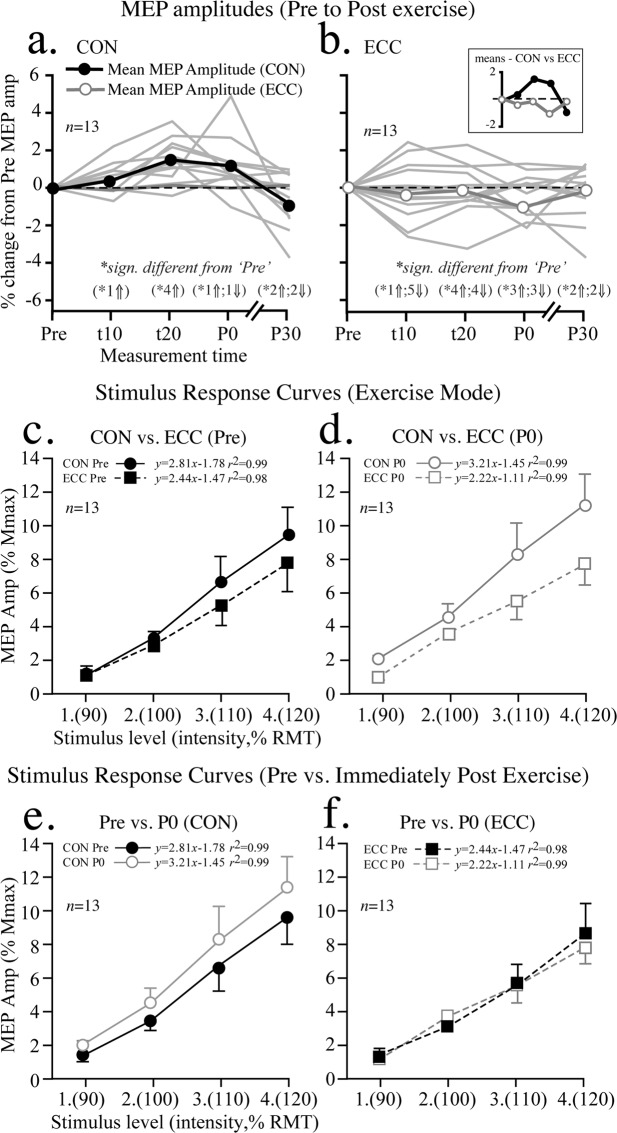
Measurements of corticospinal excitability. (**a**) CON and, (**b**) ECC mean and individual (n = 13) MEP amplitudes at all time points. Inset in (**b**) shows mean MEP amplitudes of CON and ECC conditions plotted separately for further clarity. Mean stimulus-response curves comparing (**c**) CON versus ECC at ‘Pre’; (**d**) CON versus ECC at P0; (**e**) ‘Pre’ versus P0 values for CON leg cycling; and (**f**) ‘Pre’ versus P0 for ECC leg cycling. In (**a**,**b**), Individual values (in brackets) indicate the number of participants in which MEP amplitude were significantly different from ‘Pre’ values at each time point (P < 0.05) with arrows indicating if those numbers of participants increased (⇑) or decreased (⇓) significantly with respect to Pre MEP amplitudes.

Figure 4 shows group mean (normalised to ‘Pre’ values) and individual MEP amplitudes at all time points. No significant difference existed between pre-exercise group mean RMT (*P* = 0.80, *d* = 0.06) values prior to ECC and CON cycling. Mean MEP amplitudes, normalised to pre-exercise M_max_ amplitudes (mean M_max_ amplitude: 9.8 ± 0.2 mV; range 9.2–10.1), did not differ at any time points for CON (range of *P* values = 0.15–0.1, η_p_^2^ = 0.62) and ECC (*P* = 0.93–1, η_p_^2^ = 0.17) cycling compared to respective ‘Pre’ values.

However, notable intra-individual variations in MEP amplitudes were evident when interleaved and after cycling exercise for both modes (CON and ECC). Figure 4a shows that CON cycling resulted in a trend towards increases in MEP amplitudes at 20 and P0. However, only one (10), four (20), two (P0) participant’s values were significantly different (*P* < 0.05) from ‘Pre’ values at those times. The ECC cycling mode resulted in MEP values that could be greater or lower than ‘Pre’ values; six (at t10), eight (at t20) and six (at P0) participant’s values were significantly different (*P* < 0.05) from their ‘Pre’ values at those time periods, respectively. For both CON and ECC cycling, 4/13 participants had MEP values that were greater (two) or lower (two) than ‘Pre’ values at P30 (*P* < 0.05), the other nine participants showing no significant differences.

### Stimulus-response curves

Cohort means showed that SRC were not significantly different between CON and ECC cycling before (‘Pre’, Fig. 4c) or immediately after the exercise bout (‘P0’, Fig. 4d). Within each cycling mode, there was also no significant difference between Pre and P0 measures (Fig. 4e,f).

### Changes in slope of stimulus-response curves

**Figure 5:**
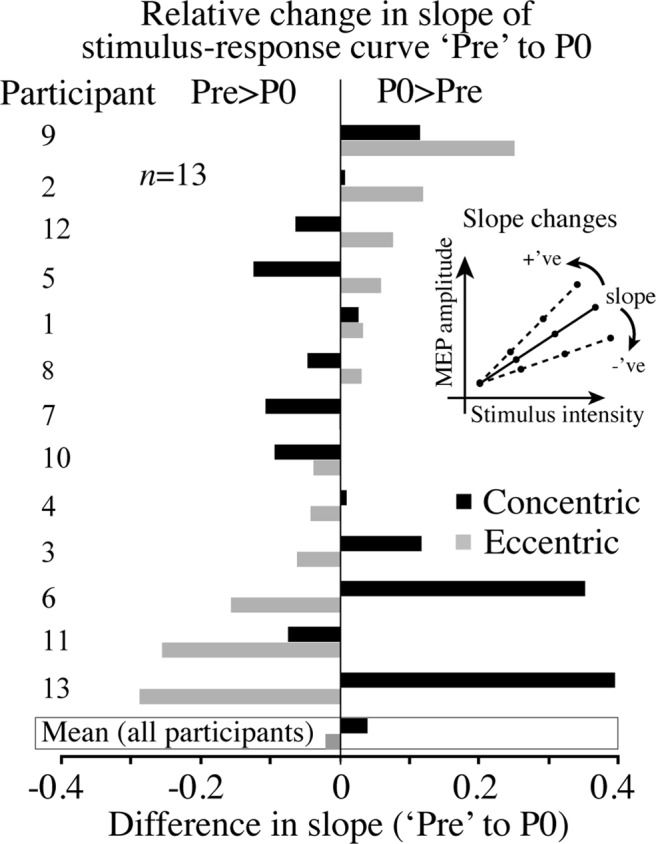
Relative changes in slope of the SRC for each participant (*n* = 13) before (‘Pre’) and immediately after (P0) exercise in each mode (CON and ECC). Values of the difference in slope of the relationship between stimulus intensity and MEP amplitude could be positive (+‘ve) or negative (−‘ve), (see inset).

Figure 5 shows relative changes in slope of the SRC between ‘Pre’ and P0 measures in the two exercise modes for all participants. In this figure participants have been ordered from the participant that showed the largest positive difference (relationship between stimulus intensity and MEP amplitude) in the ECC condition. Despite the mean slope of the entire cohort showing very little change in ECC and CON conditions, it is clear that some participants (i.e. 2, 9, 12) showed positive increases in the slope of the regression of the SRC in ECC conditions, others (i.e. 3, 6, 13) in CON conditions with a decrease in ECC conditions. These results would suggest that changes to CSE for both modes of exercise vary greatly across participants.

### Individual participant trends: power output ratios and MEP ratios

**Figure 6:**
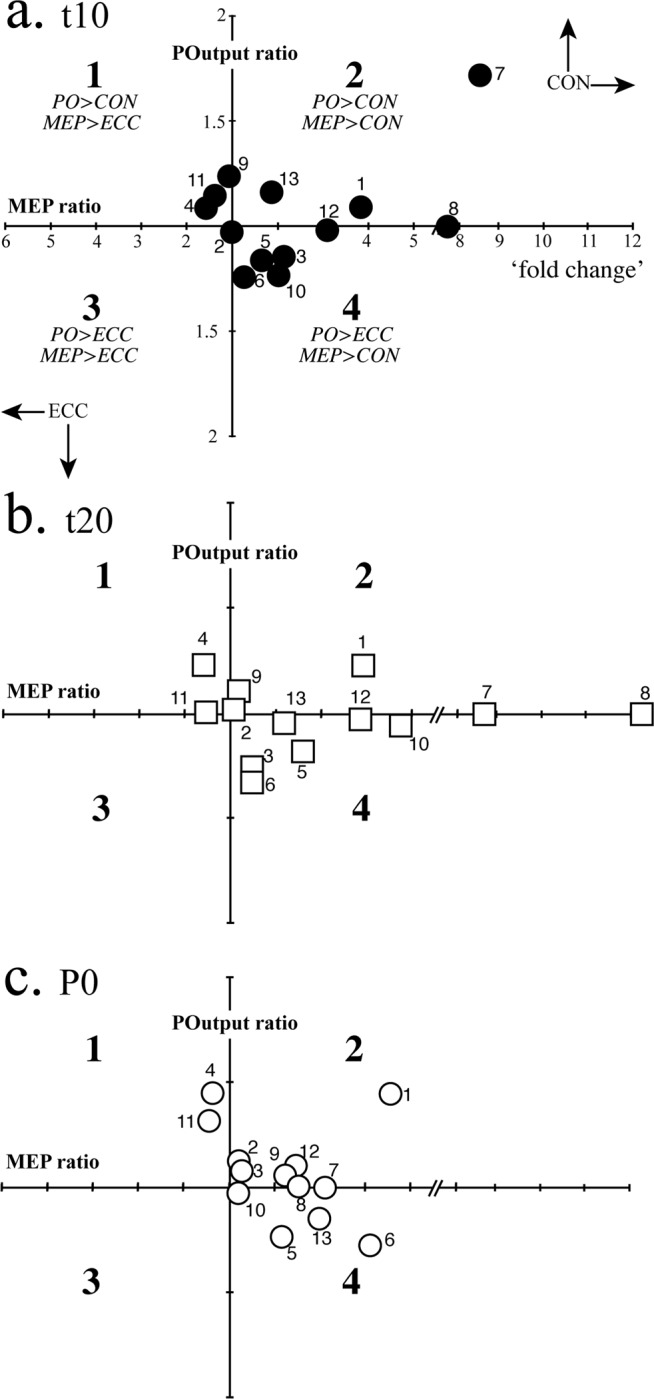
Individual (*n* = 13) ratios of MEP amplitudes (x axis) plotted against POutput (y axis) at (**a**) t10, (**b**) t20 and (**c**) P0. Axis values express ‘fold-change’; the difference between cycling modes CON and ECC in either direction (e.g., CON MEP amplitude is about 4-fold that of ECC MEP amplitude for participant 1 at t10).

Our results show that MEP amplitudes in all participants were different across ECC and CON cycling modes. To explore the relationship (if any) between the level of POutput produced in both mode and MEP amplitude, we plotted ratios of POutput and MEP change (CON: ECC ratio > 1.0 indicated greater POutput and MEP amplitudes following CON cycling, and <1.0 indicated greater for ECC cycling) for each participant at t10, t20 and P0 (Fig. 6a–c). Generally, two clusters of individuals (i.e. participants 4 and 12; 6 and 7) appear to be in contrast to the general trends. When plotting individual mean ratios of POutput (t10, t20 and P0) and MEP (10, 20, P30) amplitudes (Fig. 6), CSE appears to more responsive to CON cycling, despite two individuals showing consistently larger MEP responses to ECC cycling (i.e. participants 4 and 12) at each sampling time. Alternatively, others produced greater POutput during ECC cycling, however, show larger MEP responses for CON cycling (i.e. participants 6 and 7), while some participants (i.e. participants 13 and 11) showed adaptive changes in POutput or MEP during cycling bouts. For example, participant 13 shifted from generating greater POutput during CON cycling at t10 to generating more POutput during ECC cycling at P0.

## Discussion

This study aimed to determine how semi-recumbent ECC and CON leg cycling affects global CSE as measured in a non-exercised hand muscle. We predicted that CSE would be facilitated by CON cycling and not facilitated by ECC cycling. Our findings show that group mean global CSE as measured in the non-exercised muscle was not statistically different when interleaved and after low-intensity semi-recumbent ECC and CON leg cycling compared to ‘Pre’ exercise values. Mean stimulus-response curves compared ‘Pre’ and post (P0), showed no significant differences between CON and ECC cycling, suggesting that low-moderate intensity leg cycling may not significantly influence global CSE as assessed in a non-exercised muscle in either mode. When global CSE was plotted individually however, each participant responded differently depending on the mode of cycling, the power output produced, and the time at which the measurements were taken.

The current findings are in contrast to a previous study investigating the effect of ECC muscle contractions on global CSE assessed in a non-exercised muscle^26^. These authors measured CSE in the abductor pollicis brevis muscle pre and post uphill (CON) and downhill (ECC) treadmill running with workloads matched using heart rate. They reported a significant increase in MEP amplitude 30-min after CON and ECC treadmill running, compared to respective pre-exercise values but no differences between the two modes during exercise. Similarly, the authors reported no change in CSE measured at 5 and 15 minutes post ECC treadmill running, which is in agreement with previous studies citing a lack of change in mean CSE of the resting, non-exercised muscle^2,28^. Interestingly, this group has recently shown differences in CSE assessed between mono- and biarticular exercising muscles^38^. It should be noted however, that comparisons between CSE induced during downhill treadmill running and semi-recumbent leg cycling take place under very different conditions. Running typically involves upper and lower limb muscles which may ultimately contribute to CON activation of arm and shoulder muscles, contributing perhaps to changes in CSE. Therefore movement of the arms and legs during downhill treadmill running may greatly influence the neural coupling of the supra-spinal and spinal mechanisms compared with semi-recumbent ECC leg cycling that isolates ECC contractions to the leg muscles. As such, semi-recumbent ECC cycling provides a means of determining the neural control mechanisms governing rhythmic ECC movement. Eccentric cycling may involve greater supra-spinal control^25^ requiring less spinal CPG activation to non-exercised muscles therefore reducing the strength of the neural coupling between the upper and lower limbs. This may explain why no change in global CSE was evident at interleaved times and following semi-recumbent ECC leg cycling in the current study. However, given that CSE did not differ for CON cycling, the low-intensity of the exercise may have had a substantially greater contribution to the lack of exercise-induced CSE.

As indicated by MEP amplitudes measured pre and post ECC and CON leg cycling, the results of the present study show that, regardless of mode of contraction (i.e. CON/ECC cycling) low-moderate intensity semi- recumbent cycling exercise does not appear to significantly alter group mean values of global CSE. Our findings support those previously reported^2^, but differ from a number of studies that have observed increased CSE as seen through the activity of non-exercised upper limb muscles following low-intensity CON leg cycling^4,28,39^. However, among certain individuals we did observe significant increases in MEP amplitude for low-intensity CON cycling (Fig. 4a). Also, in our experiments, global CSE did return to near pre-exercising (resting) values by at least 30 minutes post ECC and CON cycling, confirming the relatively rapid decay of CSE after the cessation of exercise^40,41^. Nevertheless, despite similarities in group mean responses, select individuals varied with respect to global CSE assessed for semi-recumbent ECC and CON leg cycling (Figs. 4 and 5). A number of participants did show unexpected increases in MEP amplitudes interleaved for ECC leg cycling, suggesting submaximal ECC cycling may increase CSE in a resting, non-exercised muscle in at least some persons. Furthermore, some participants showed increased CSE of the resting, non-exercised muscle in the ECC cycling condition, in the form of increased slope of stimulus-response curves (Fig. 5) and MEP ratios (Fig. 6), the latter despite power output ratios favouring CON cycling. Such a change in slope of the stimulus-response curve after either mode of exercise may indicate plasticity of the corticospinal pathway induced by the respective mode of exercise^42^. Alternatively, other participants produced fluctuating power outputs across the 30 minutes of cycling, varying between ECC and CON cycling at t10, t20 and P0 (Fig. 6). However, these participants showed MEP ratios favouring CON cycling, further indicating that CON muscle actions may have had greater excitatory influence on global CSE. These results suggest that an individual approach to determining the effects of ECC exercise on CSE is likely to produce the most meaningful outcomes. We should not rule out the possibility however, that the variations in individual values may have been due to the inherent variability of MEPs produced using TMS, therefore any conclusions made from single participant data, especially using 5 MEPs at each measurement time should be interpreted with caution. Ideally, repeated experiments in the same cohort and a greater number of MEPs in each condition would attenuate this.

The obvious inter-individual differences seen across the cohort may in fact have been a result of the most obvious shorter period of familiarisation in ECC cycling (5 min) of the participants compared to their evidently already familiarised CON cycling pattern. This is particularly evident in participant 1 (Fig. 3b) who showed significant variability in, and lower overall values of ECC PO even after 30 min of cycling. Indeed, this participant became more concentrically-dominant in terms of both MEP and PO ratios as the experiment progressed (compare Fig. 6a–c). On the contrary, participant 5 (Fig. 3c) was able to maintain a more uniform and less variable PO throughout the experiment, albeit slightly lower than CON PO. Interestingly, this participant moved further into quadrant 4 of Fig. 6 (greater MEPs after CON cycling, but with a PO that was greater for ECC cycling). It may be therefore, that certain persons are able to familiarise more quickly to ECC cycling than others. This might involve adopting strategies directly from learned motor patterns of CON cycling, particularly anticipating the movement of the pedals backwards and producing motor activity that anticipates the application of force at the limb. In other words, in order to eliminate the contamination of an ECC dataset by learned CON motor patterns, eccentric cycles should incorporate a trip system whereby any anticipation of backward pedal movement using concentric muscle contractions cannot occur. A robust measure of familiarisation to semi-recumbent ECC cycling should also be incorporated into a study before any neurophysiological measures are taken. An example of this would be to calculate the minimal detectable change^43^ that involves determining the difference between true changes with motor learning of a task and random error.’

To a certain extent therefore, our results that certain individuals increased (significantly so) in global CSE within the ECC cycling condition would not support the premise in the Introduction that eccentric muscle contractions are not associated with a facilitation of CSE. This may have occurred through the known inhibition of Ia afferents, as well as recurrent inhibition to regulate gain at the spinal level^20,21,44^. However, the lack of measurement techniques employed in the present study that may have eluded to sub-cortical or spinal mechanisms at play limits any interpretation of the mechanisms that may have contributed to changes in global CSE following ECC cycling in the current study. Furthermore, the prescribed moderate-intensity of exercise adopted may have failed to induce acute changes in the neural response governing rhythmic muscle contractions resulting in minimal effects on CSE^45,46^. As a result, group mean corticospinal responsiveness may be influenced by exercising intensity, therefore the precise cortical, sub-cortical and spinal mechanisms underlying ECC muscular contractions, particularly for semi-recumbent ECC cycling require further investigation using higher intensity exercise protocols, as potential benefits of ECC exercise may be useful for neurological rehabilitation^47^.

As with any TMS study, the current one would have benefited from the use of neuro-navigation to more accurately reproduce MEPs from the originally determined hotspot, particularly when using a cross-over study design. However, several previous studies looking at MEP responses following aerobic exercise have reported significant findings without using specific neuro-navigation equipment^2,28,41^. Additionally, this study would have been better served using a consistent ISI, a single-pulse stimulus intensity of 120% RMT and a stimulus-response curve protocol that included stimulus intensities at 130 and 140% RMT. It could be that potential changes to CSE in a non-exercised muscle, resulting from ECC and/or CON, were missed by not sampling MEPs at higher stimulation intensities. Finally, measuring M_max_ ‘Pre’ and post (P0) exercise would have provided a more consistent indication of relative changes to CSE of the resting, non-exercised muscle following ECC and CON cycling. However, previous studies have reported conflicting findings when measuring M_max_ amplitude pre and post CON cycling in a non-exercised upper limb muscle at low-moderate^2,37^ and high intensities^36,48^, indicating variability for M_max_ amplitude, at least in response to CON exercise. Furthermore, when measured in an exercised muscle, no change in M_max_ amplitude was cited following lengthening contractions of the peroneal muscle^49^. Therefore, it is reasonable to normalise MEPs collected *interleaved* and *following* ECC and CON cycling to pre-exercise M_max_ in the current study. However, we do acknowledge that M_max_ is a measure that is sensitive to changes in temperature and conductance of electrodes that alter M_max_ recordings with a session^50,51^.

In conclusion therefore, despite the increasing use of semi-recumbent ECC leg cycling within rehabilitation, the influence of ECC leg cycling on the excitability of the corticospinal pathway remains largely unknown. We have shown that group mean values of global CSE, measured from a non-exercising hand muscle, are not significantly different between ECC and CON cycling *interleaved* or *following* the exercise executed at comparable perceived workloads. Based on our findings, continued investigation of the influence of semi-recumbent ECC leg cycling on CSE measured in both exercising and non-exercising muscles and sub-cortical or spinal mechanisms involved are required to more fully understand the neural control strategies underlying this mode of contraction, as well as any potential neuromuscular benefits accompanying continued adaptation to ECC cycling. Most importantly, the findings of this study suggest that any future studies need to consider neurophysiological measures in relation to physiological variables produced during the exercise, such as power output, on an individual basis as likely both evolve with experience of the exercise, most notably during the novel ECC mode. However, they can only do so after experimenters are sure of a familiarised motor pattern of semi-recumbent ECC leg cycling.

## Data Availability

We attest to making our data available upon request.
